# Co-evolutionary dynamics of mammalian brain and body size

**DOI:** 10.1038/s41559-024-02451-3

**Published:** 2024-07-08

**Authors:** Chris Venditti, Joanna Baker, Robert A. Barton

**Affiliations:** 1 School of Biological Sciences, University of Reading, Reading, UK; 2 Department of Anthropology, Durham University, Durham, UK

**Keywords:** Evolution, Phylogenetics, Coevolution

## Abstract

Despite decades of comparative studies, puzzling aspects of the relationship between mammalian brain and body mass continue to defy satisfactory explanation. Here we show that several such aspects arise from routinely fitting log-linear models to the data: the correlated evolution of brain and body mass is in fact log-curvilinear. This simultaneously accounts for several phenomena for which diverse biological explanations have been proposed, notably variability in scaling coefficients across clades, low encephalization in larger species and the so-called taxon-level problem. Our model implies a need to revisit previous findings about relative brain mass. Accounting for the true scaling relationship, we document dramatically varying rates of relative brain mass evolution across the mammalian phylogeny, and we resolve the question of whether there is an overall trend for brain mass to increase through time. We find a trend in only three mammalian orders, which is by far the strongest in primates, setting the stage for the uniquely rapid directional increase ultimately producing the computational powers of the human brain.

## Abstract (web summary)

Analysis of mammalian brain and body mass reveals a curvilinear relationship contrary to assumptions of log-linear power laws. As mammals grow larger, increases in brain mass compared to body mass diminish.

## Main

For the past 100 years, it has been routine in comparative biology to describe the relationship between brain and body mass in mammals by the power law: *y* = *ax*^*b*^, where *y* = brain mass, *x* = body mass, *a* = the intercept and *b* = the allometric coefficient. Conventionally, this is expressed in logarithmic form with the assumption that this linearizes the relationship between brain and body mass: log(*y*) = log(*a*) + *b*log(*x*). The value of the allometric coefficient is then often taken to reflect a fundamental underlying scaling rule, but debate on its value and biological relevance has been rife. Prominent claims, based on different theoretical postulates, are that it reflects surface–area relationships, predicting a 0.67 exponent, or the scaling of metabolic costs of the brain, predicting a value of 0.75 (refs. ^1–3^). It has become apparent, however, that using this approach with large datasets reveals puzzling heterogeneity in the scaling exponent, both across taxonomic groups (for example, between different orders) and at different taxonomic levels (for example, families versus orders). Recent studies of both birds and mammals^3–5^ document significant differences in both the exponent and the intercept between orders, suggesting that no single biological process regulates the relationship between brain and body size. The scaling of brain to body size has also been found to vary systematically across taxonomic levels, with slope values being higher among higher taxonomic levels such as between species within genera compared with genera within families (for example, ref. ^5^). A variety of explanations for this ‘taxon-level problem’ have been proposed^6,7^, including the idea that brain mass lags behind body mass when there is strong selection on body size causing rapid evolutionary change, with brain size gradually catching up over longer evolutionary periods^8^. However, direct tests of this hypothesis have found no evidence for lag^9,10^.

Whatever the explanations for these effects, they have consequences for understanding the biological significance of relative brain size, along with how it has evolved over time and across species. Relative brain size is frequently assumed to reflect selection on cognitive capacities^11,12^ but, because of the taxon-level effect, when estimated across higher taxonomic units, relative brain size will be smaller for large-bodied species than their smaller close relatives; this raises doubts about what such estimates mean for cognitive function and further suggestions as to the most biologically meaningful measure of relative brain size^13^. Similarly, heterogeneity in exponents across different mammalian orders implies that there are yet undiscovered reasons for diversity in the scaling rules and that relative brain sizes estimated without taking such heterogeneity into account risk conflating different sorts of effects.

Here we re-examine such questions by asking a fundamental question: is the assumption of log-linearity correct, and if not, what are the consequences for understanding brain size evolution? A new generation of phylogenetic comparative methods allow us to test this fundamental question while simultaneously testing for significant variation in the rate of brain size relative to body size evolution across the mammalian tree of life. Such analyses have the potential to uncover long-term evolutionary trends, such as the longstanding Marsh–Lartet rule^13,14^. The Marsh–Lartet rule posits a trend towards increasing relative brain mass through time in mammalian evolution. Under this model, we would expect to see relative brain size increase more than expected if brain size predominantly reflects body mass evolution, which we also expect to increase through time according to the well-known Cope’s rule^15^.

## Results and Discussion

### Characterization of the mammal brain–body mass relationship

**Fig. 1:**
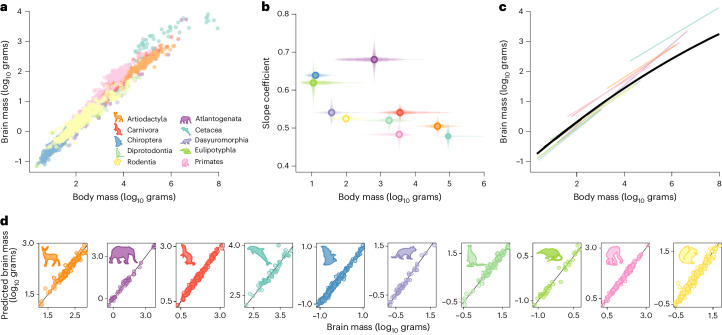
The curvilinear relationship of the BBM relationship across mammals. **a**, Brain and body size data used in this study coloured by mammalian taxonomic group. **b**, Slope coefficient (and percentiles of posterior distributions plotted as transparent lines) for each taxonomic group from **a**, plotted against body mass (percentiles of body mass range plotted as transparent lines) for each group. **c**, The median model prediction for our curvilinear BBM relationship across mammals from the variable rates regression model (black) with the variable-slope model predictions for comparison. **d**, Actual brain mass against predicted brain mass from the curvilinear model, highlighting the accuracy of the fit to the data. In all panels, points and lines are coloured according to orders as shown by representative silhouettes (see legend, not to scale).

**Extended Data Fig. 1:**
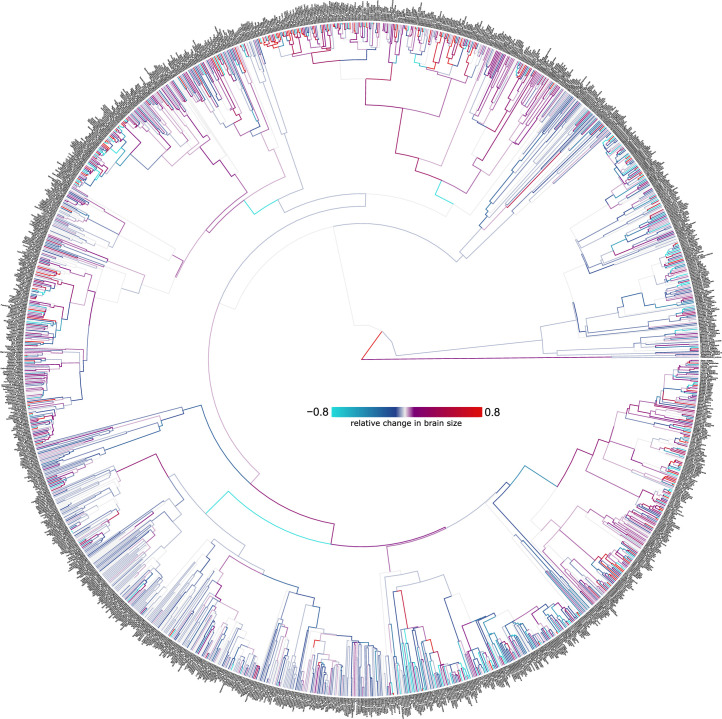
Magnitude of brain and body size changes observed across the entire mammalian phylogeny. The branches of the mammalian phylogeny (N = 1436) (proportional to time in millions of years) are coloured by the estimated difference in the standardized magnitude of change in brain mass compared to body mass on a log-log scale (Z_brain_-Z_body_).

We use a phylogenetic approach applied to a comprehensive dataset of brain and body masses (*n* = 1,504, Fig. 1a) spanning the mammalian radiation to flexibly characterize the underlying brain–body mass (BBM) relationship while simultaneously detecting rapid increases or reductions in the rate of relative brain mass evolution^16–18^ (Methods). The BBM relationship across diverse animal clades, such as the mammals, has usually been studied by fitting models with multiple slopes and intercepts to account for differences between clades (for example, refs. ^4,19–21^). In congruence with these previous studies, a multiple-slopes model (in which a separate slope is estimated for each order) demonstrates that the BBM relationship varies between orders (Fig. 1a). However, the variability in slopes across mammals appears to be a mass-dependent phenomenon. Across the range of mammalian body mass, there is a negative correlation between the slope of a mammalian order and its average body mass (*ρ* = −0.63, *P* = 0.049, Fig. 1; *ρ* = −0.82, *P* = 0.006 after excluding Atlantogenata which, despite a large mean body mass, spans the extremes of mammalian body size range—over 5 orders of magnitude). This negative correlation is even more pronounced across mammalian families (*ρ* = −0.56 *P* < 0.001, Extended Data Fig. 1). There have been some hints in the literature pointing to a potential mass dependency or curvature in the BBM relationship^22–25^. However, to the best of our knowledge, this has neither been tested against a multiple-slopes model nor in a phylogenetic context and has been ignored by the vast majority of comparative studies. Thus, our results suggest that the variability in slopes previously attributed to different patterns of selection or scaling rules in different clades^3–5^ may simply reflect failure of linear models to adequately account for the effect of body mass.

To explicitly test this hypothesis, we fit a curvilinear relationship (second-order polynomial). Using the proportion of the posterior distribution of the estimated parameter crossing zero (*P*_x_) to assess significance (a variable is significant when *P*_x_ < 0.05), we find significant curvature in the BBM relationship (*P*_x_ = 0, median *β* = −0.019, median *R*^2^ = 0.89; Fig. 1c). We compared model fits using Bayes Factors (BF); where BF > 2, it is considered support for one model over another (see Methods for more details). We find that our single-slope curvilinear mass-dependent model fits significantly better than the multiple-slope linear model (BF = 155.85, see Supplementary Information): as mammals increase in mass, the rate at which brain mass increases with body mass decreases, even after logarithmic transformation. This result is robust to intraspecific variation (tested using between 1 and 59 individuals per species, see Methods). Our results show that the brain size of the largest mammals changes by ~44% less per unit body mass than that of the smallest mammals (Supplementary Fig. a1). Interestingly, there is no significant variation in the curvilinear relationship among orders (Supplementary Information), implying that a single scaling rule adequately accounts for the BBM relationship. While across the range of our data the second-order polynomial model fits well with no systematic bias (Fig. 1d), it might be argued that it is more appropriate to fit a power curve to the log-log data which asymptotes. With this in mind, we conducted two additional sets of analyses using power curves (Supplementary Information). The predictions for these are almost indistinguishable from the second-order polynomial (Supplementary Fig. a1). Our results demonstrate that the BBM relationship does not conform to the established theoretical power-law expectations given surface–area relationships and metabolic costs^1–3^.

These results reveal that phenomena such as the previously reported variability in the slopes and intercepts of the log-linear BBM relationship across mammalian orders^4^ and apparent evolutionary lags in brain mass relative to body mass are explained exclusively as mass-dependent effects rather than taxon-specific patterns of brain evolution^24,26^. With this mass dependence in mind, we can shed new light on the well-known ‘taxon-level effect’^6^ in the BBM relationship among mammals. Although many ideas to explain the taxon-level effect have been proposed, none have proven robust, and for that reason, the phenomenon and its causes remain contentious^5,6,24,26,27^. However, our results show that the covariance of the BBM relationship changes with body size. We therefore suggest that the apparent taxon-level effect emerges simply as a side-effect of the curvilinearity of the BBM relationship combined with the trend for increase in body mass over time, known as Cope’s rule^15^. Strong evidence from both the fossil record^28^ and extant species^16,29^ support Cope’s rule in mammals. Given this pattern, linear regression coefficients will inevitably be shallower in more closely related species compared with more distantly related species. This is purely because more distantly related species are more likely to have branches that span into deep time such that the evolutionary signature of Cope’s rule should be stronger. Our results therefore suggest that more complex evolutionary explanations for the taxon-level effect, involving decoupling of brain and body mass and evolutionary lags for example^24,26^, are unnecessary.

### Rates of brain size evolution across the mammal radiation

**Fig. 2:**
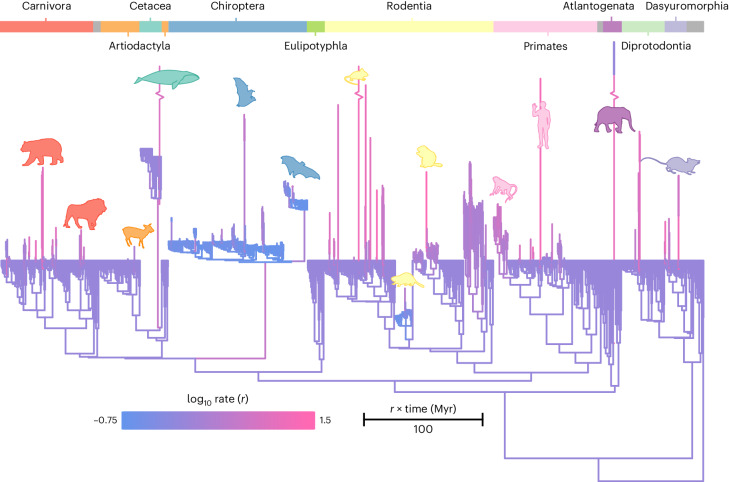
Rates of relative brain mass evolution. The mammal phylogenetic tree used in this study where branches are coloured according to the (log_10_) rate of relative brain mass evolution (see scale bar). The original branch lengths of the tree, measured in time (Myr) have been compressed and stretched by the median rate of evolution such that branch length = *t* × *r* (see scale bar). Long branches represent lineages where relative brain size evolution was accelerated. Mammalian taxonomic groups are represented by the coloured bars along the top of the figure. Selected branches with relatively high (and low) rates of evolution are highlighted by representative silhouettes using the same colour scheme (not to scale). Three branches have been broken for aesthetic purposes as they all had very high rates on relatively long branches: the branch leading to Temminck’s mouse (*Mus musculoides*, median *r* = 20.72 and *t* = 9.52); the branch leading to the bowhead whale (*Balaena mysticetus*, median *r* = 9.32, *t* = 25.9) and the branch leading to the two extant elephant species (median *r* = 16.41, *t* = 40.43).

After accounting for the mass-dependent scaling of the BBM relationship, there is substantial variation in evolutionary rates (Fig. 2). If relative brain mass predominantly reflected body mass evolution (for example, refs. ^4,24,30,31^), then we would expect little to no rate heterogeneity (that is, mammal brain size would simply be a consequence of body size evolution). However, we find that all orders show branches where the rate of relative brain mass evolution is increased; this is most pronounced in Primates, Rodentia and Carnivora (Fig. 2). Although there is a high rate on the branch leading to Chiroptera (Fig. 2), bats as a clade tend to have a very low rate of relative brain mass evolution (~2.5 times lower than the mammalian background rate), which might indicate an evolutionary constraint associated with flight. Bat clades with significantly elevated rates do not appear to be united by any obvious factors. Several ecological factors such as diet^32^ or hibernation^33^ have been proposed to drive brain size in Chiroptera, yet it remains to be formally evaluated whether these or other factors may give rise to the variable rates we observe. Confirming previous suggestions^34^, the rate of relative brain mass change we observe on the branch leading to humans is extremely high, with a median rate 23 times higher than the mammalian background rate.

### Evolutionary trends

**Fig. 3:**
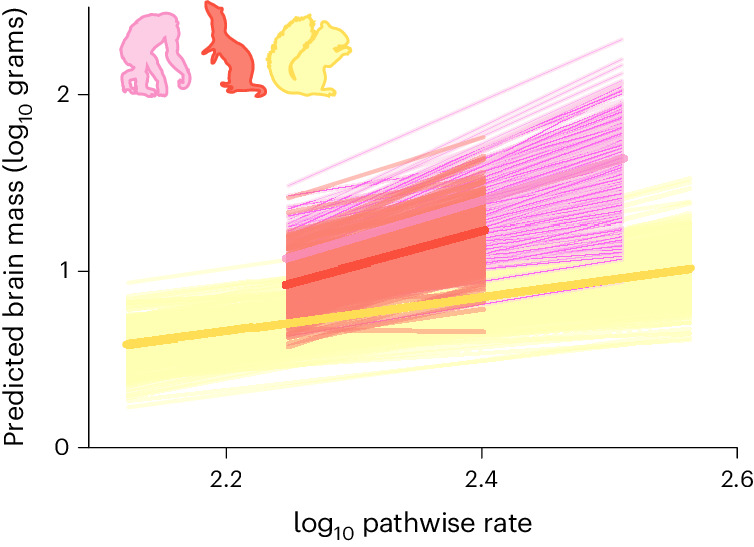
Trends towards increasing brain mass through time. Posterior distributions (transparent lines) and medians (solid lines) of the model predictions demonstrate the trend in three mammalian orders (rodents, yellow; carnivores, red; primates, pink). Silhouettes indicate the relevant taxonomic groups (see Fig. 1) and are not to scale.

Analyses of rate heterogeneity similar to those we use here introduce meaningful variation into the branch lengths of a phylogeny (Fig. 2). This makes it possible to study evolutionary trends in trait (or relative trait) evolution through time (for example, refs. ^16,35,36^). Longer branches represent an increase in the rate of evolution probably owing to the influence of selection^36,37^, that is, they have undergone more relative brain mass change than would be expected given their length in time. The sum of all rate-scaled branches along the evolutionary path to each species (pathwise rate) can therefore be used to measure the total amount of evolutionary change that a species has experienced during its history^16,36^: Species with longer pathwise rate values have experienced more relative brain size change throughout their entire evolutionary history. If pathwise rates are correlated with brain size (in either direction), such change has been predominantly directional and the only way that this could occur is via an evolutionary trend: repeated, rapid changes towards larger (or smaller) size throughout a clade’s evolutionary history^16,35^. With this in mind, we use Bayesian phylogenetic regression models to determine whether there have been any long-term evolutionary trends in relative mammalian brain mass through time (for example, towards larger or smaller mass). Across all mammals, we find a significant increase in relative brain mass with pathwise rate (*β* = 0.906, *P*_*x*_ = 0.000). However, when we allow the slope of the relationship between relative brain mass and pathwise rate to vary among orders (that is, test to see whether the trend is the same among mammalian orders), we find a significant trend in only three orders: rodents (*β* = 0.998, *P*_*x*_ = 0.002), carnivores (*β* = 1.845, *P*_*x*_ = 0.000) and, most strikingly, primates (*β* = 2.074, *P*_*x*_ = 0.000) (Fig. 3). No such relationship is found in any other order (all *P*_*x*_ > 0.1) or across all remaining mammals (*P*_*x*_ = 0.331), hence the relationship observed across all mammals is driven by the trend in these three groups. This demonstrates that the Marsh–Lartet rule^13,14^, proposing a trend for relative brain mass to increase throughout mammalian evolution, is not a general mammalian phenomenon but a particularity of only a few orders.

**Fig. 4:**
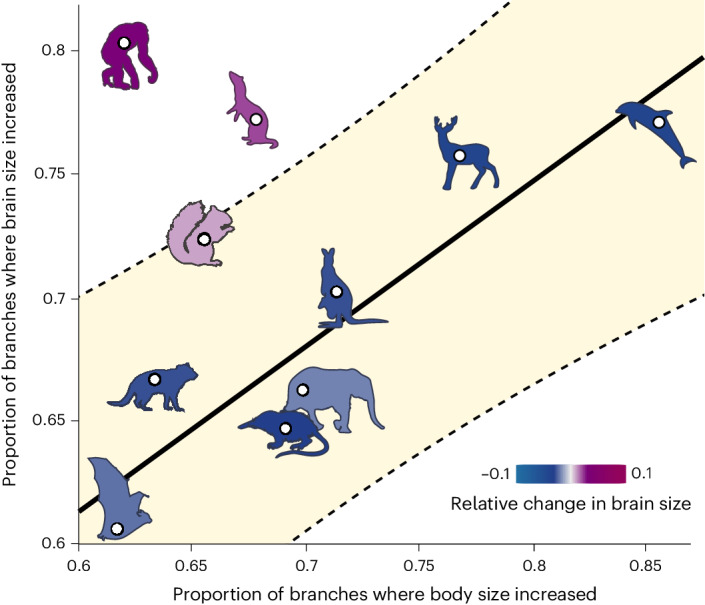
Proportion of branchwise brain and body size increases. The proportion of branches where brain mass increased compared with the proportion of branches where body mass increased is plotted per taxonomic group. The fitted line and 95% prediction intervals (shaded) highlight primates and carnivores as outliers. A representative silhouette for each order (see Fig. 1) is shown and coloured according to the median amount of total relative change in brain size for that order (estimated difference in the standardized magnitude of change in brain mass compared to body mass, *Z*_brain_−*Z*_body_).

**Fig. 5:**
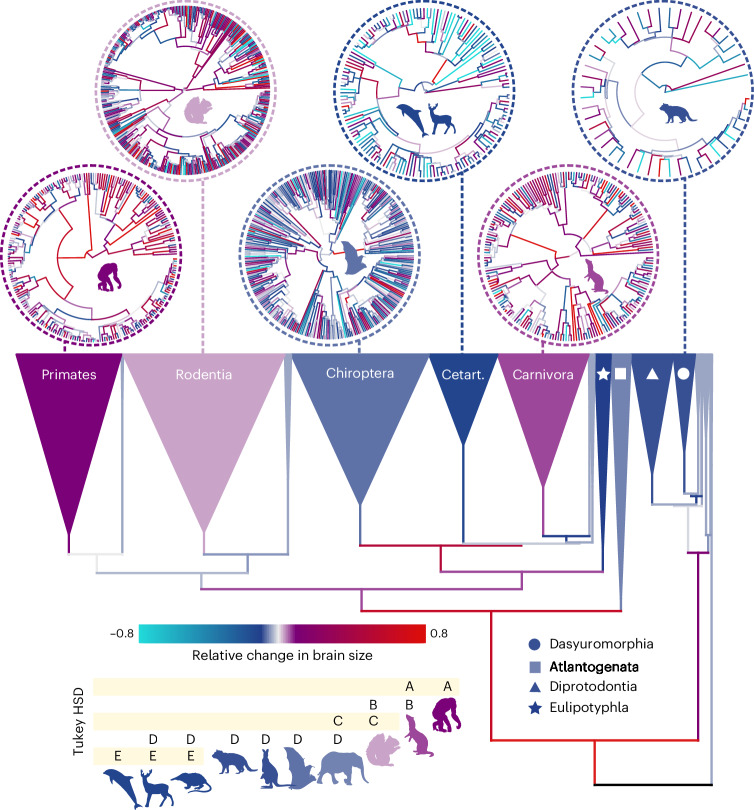
Magnitude of branchwise brain and body change. Phylogenetic branches (and collapsed clades) are coloured by the estimated difference in the standardized magnitude of change in brain mass compared to body mass (*Z*_*brain*_−*Z*_*body*_). Clades are coloured according to the median value. Inset shows the result of a post hoc Tukey HSD test, in which each taxonomic group is assigned a letter (A–E) on the basis of its similarity to other groups (that is, groups that share a letter are not substantially different from one another). We show zoomed in circular branchwise phylogenies for six representative clades (Primates, Rodentia, Chiroptera, Cetartiodactyla, Carnivora and Dasyuromorphia) to illustrate how branchwise brain and body change have played out in these groups and how this links to the clade-level values in the collapsed phylogeny and Tukey tests. A full version of this tree can be found in Extended Data Fig. 2.

The unique trajectory we reveal in primates is apparent if we simply compare the inferred body mass and brain mass change along the branches of the phylogenetic tree for each order of mammals we study (Fig. 4, estimated using the parameters of our models; see Methods). When the proportion of branches where we observe brain mass increase are compared with the proportion where body mass increases, only the primates and carnivores are clear positive outliers. Primates are the extreme case in which over 80% of branches underwent a brain mass increase, compared with under 65% where body mass increased (Fig. 4). We can further examine the unique nature of this trend when we compare the standardized magnitude of change in brain compared to body. We conducted an analysis of covariance accounting for ancestry, comparing the reconstructed brain change along each branch in each of our orders, accounting for body size change (*P* < 0.0001, Fig. 5). The inset of Fig. 5 shows the result of a post hoc Tukey HSD (honestly significant difference) test, which demonstrates (along with the coloured radial trees in Fig. 5) that among mammals, primates have the highest relative change in brain size followed by carnivores and rodents. Because the trend in relative size is clearly not driven by body size change, we can therefore exclude the hypothesis that large relative brain size in primates predominantly reflects reductions in body size^24^.

Natural selection has decoupled brain and body mass evolution in primates to a unique extent, producing sustained and directional increase in relative brain mass for over 55 million years (Myr). This trend set the stage for rapid increase in hominins, leading ultimately to modern humans’ unprecedentedly large brains^34,38^. Hence, the emergence of human-like cognitive capacities was facilitated by a shift in the fundamental pattern of brain evolution at the origin of the primates. An explanation for this distinctive pattern in primates, whether in terms of the release of a constraint or an adaptive shift that initiated escalating feedback between brain and behaviour, would be a notable contribution to biology. Candidates might include sociality^39^, diet^40^, unusual patterns of maternal investment facilitating extended brain growth^1^, or the advent of visuo-motor control of the forelimb associated with stereoscopically guided grasping and manipulation and a unique pattern of connectivity between eye and brain^41–43^. Future studies testing these hypotheses should now account for the curvilinear relationship we have demonstrated.

### New directions

Our results raise important questions for future research: (1) Why does a curvilinear relationship exist? and (2) Do any non-mammalian clades show similar size dependency? With respect to the former, if the mass dependency we observe reflects how neural function is conserved across the range of mammalian body sizes, we might expect factors such as synapse density, cell number, cell size and connectivity to be important in the scaling of the BBM relationship^44^. We tested to see whether the efficiency of brain connectivity (quantified by using the species-level connectivity network’s mean-short path, MSP^45^, which is the minimum distance that each neuron is away from all other neurons and can be interpreted as a measure of total brain network communication) explains the curvature we observe. In a subset of our data (*n* = 104) for which MSP is available, we found that MSP was not significant in our phylogenetic model (*P*_*x*_ = 0.37) and did not diminish the curvature we observe. Likewise, in the same subset of species (*n* = 104), we were able recover the same curvilinear relationship in both grey (*P*_*x*_ = 0.013) and white (*P*_*x*_ = 0.004) matter of the brain (measured at species level), which we would not expect to be the case if the scaling of axon size and/or myelination explained the curvilinearity. Taken together, these preliminary results indicate that connectivity may not be the driving force behind the curvature we reveal.

Another possible explanation of our results might come from considering the energy balance in the evolution of the brain. Brain tissue is notoriously energetically expensive and basal metabolic rate (BMR) has been shown to be associated with brain size, even after accounting for body mass (for example, ref. ^46^). If large species cannot sustain the high cost of their large brains, we might expect that if we include BMR in our model, that would explain the size dependence in the BBM relationship. We test this by incorporating BMR data for a subset of 572 mammals into our quadratic model. We find that BMR does significantly contribute to our model (*P*_*x*_ < 0.001), that is, it has a significant effect on brain size beyond body size. However, it does not diminish the curvature we identify (*P*_*x*_ = 0.033), suggesting that balancing energy resources in large-bodied species is unlikely to be driving the mass-dependent relationship.

**Extended Data Fig. 3:**
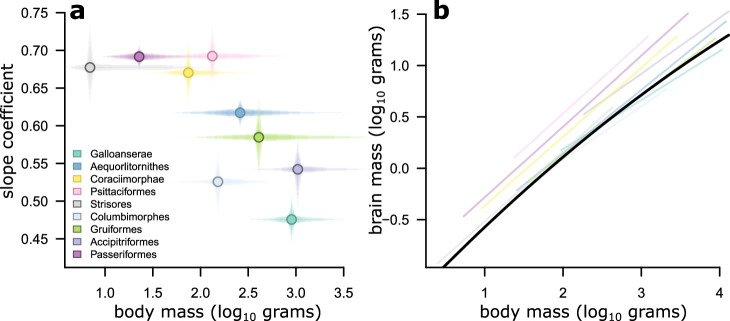
Curvilinear relationship between brain and body mass across birds. **a**, Slope coefficients (and percentiles of posterior distributions plotted as transparent lines) for each major avian group plotted against the average body mass for that group (percentiles of body mass range plotted as transparent lines). Slope coefficients were estimated from a multiple-slopes variable rates regression model run identically to the multiple-slops model presented for mammals. **b**, The median model predictions for our curvilinear BBM relationship across all birds from the variable rates regression model (black) compared to the multiple-slope model predictions. The curvilinear model is statistically supported.

Is there something about the architecture of the mammalian brain that dictates its log-curvilinear scaling, or do other major vertebrate clades show similar size dependency? Birds are the only clade for which comparable brain and body size data are available to test the generality of our findings. We matched 1,182 avian species brain and body masses^47^ to a comprehensive bird phylogeny^48^ spanning the avian radiation. We find striking similarities in the results of birds to what we find in mammals. Our results show that a model that allows the BBM relationship to vary among avian orders (multiple-slope) fits our data better than a single BBM relationship (single-slope) in a log-log space (BF = 12.96). We also see the significant negative correlation between the slope of avian orders and its average body mass (*ρ* = −0.76, *P* = 0.0178, Extended Data Fig. 3). As expected from mammals, a curvilinear mass-dependent model fits the bird data significantly better than the multiple-slope model (BF = 34.24, Extended Data Fig. 3). The mass dependency we identify here is, therefore, ubiquitous among endotherms. Whatever explains the phenomenon, it is thus not something specifically related to the architecture of mammalian brains, requiring more general explanations to be sought.

## Conclusions

Our results have profound implications for the study of brain size evolution. By simultaneously explaining multiple statistical phenomena reported on the basis of linear models, our results resolve several debates about the co-evolutionary dynamics of mammalian brain and body size. This obviates the need for special explanations proposed for each individual phenomenon, including the taxon-level effect, lag in brain mass relative to body mass evolution, differences in brain–body intercepts (‘grade shifts’) and differences in slopes^4,21,49,50^. Previous conclusions regarding the evolutionary relationship between brain and body mass, how it changes through time and/or among phylogenetic groups, estimates of relative brain mass in particular taxa and methods for deriving them (for example, refs. ^4,13,31^) need to be re-evaluated in light of our findings. In particular, our results can explain why estimates of encephalization are biased with respect to mass, being lower than expected in large-bodied animals^13^. In addition, our results suggest that correlates of relative brain mass evolution (recently reviewed in ref. ^51^), whether behaviour, ecology development or life history, will need to be re-assessed after accounting for the curvilinearity in the BBM relationship.

Beyond brain evolution, our results contribute to emerging evidence of curvilinear mass dependence in allometric relationships across diverse phenomena and species (for example, refs. ^52–57^). Taken together, these results should shift researchers’ attention away from assuming the ubiquity of simple power-law associations. Seeking both the theoretical and empirical underpinning for curvilinear relationships across species will probably lead to major contributions across biology.

## Methods

### Data and phylogenetic tree

For the mammals, our primary dataset comprised 1,504 species-level brain and body masses taken from the literature (Supplementary Table 1). We collated measurements from various sources, prioritizing those that report brain mass over brain volume (or converted brain volume) and preferring datasets where body mass was measured from the same individuals as brain size. In other cases, values were reported as species averages (see original sources for more details); we calculated weighted means where possible. All data and original sources are reported in our Supplementary Information. The phylogenetic tree was taken from the time tree of life^48^.

For the birds, we used a published dataset of brain and body masses^47^. We matched this data to the time tree of life^48^ for a total of 1,253 species. In some cases, multiple data points were found for a single tip in the tree (for example, *Phaps* is represented at genus level in the time tree of life, but there is brain size data for multiple species within the genus). In these cases, a single datapoint was selected at random to represent the tip (although our results are identical if we use all available data). Using the taxonomic groupings from Prum, the dataset was reduced to 1,182 species—those in taxonomic groups large enough to estimate separate slopes (*N* > 20; see below).

Mammalian connectivity (MSP), grey matter and white matter data were taken from ref. ^45^. We matched 104 species to our mammal brain and body size data. We obtained basal metabolic for 572 species found in our mammal brain and body size data from the literature.

### Rate heterogeneity

To determine the extent of variation in the rate of brain mass evolution after accounting for body mass (relative brain mass evolution), we used the ‘variable rates regression model’^17,37^. This Bayesian Markov chain Monte Carlo (MCMC) regression technique acts to estimate the rate of evolution in the phylogenetically structured residual error of a regression model^17,37^. The model simultaneously estimates a Brownian motion process (background rate, $\,{\sigma }_{b}^{2}$) with a set of rate scalars *r* defining branchwise shifts (identifying branches evolving faster (*r* > 1) or slower (0 ≤ *r* < 1) than the background rate). We then multiplied the original branch lengths (measured in time) by the corresponding *r* for each branch, resulting in a scaled phylogeny where longer branches (compared to their original length in time, *r* > 1) indicate faster rates of morphological evolution and shorter branches (0 ≤ *r* < 1) have slower rates. These branch-specific scalars therefore optimize the fit of the phylogeny to the underlying background rate ${\sigma }_{b}^{2}$ given the inferred phenotypic change along each branch.

To identify evidence for rate heterogeneity, we used BF = −2log_*e*_(*m*_1_/*m*_0_), comparing the marginal likelihood of our variable rates model (*m*_1_) to that of a model with a single underlying ${\sigma }_{b}^{2}$ (*m*_0_). BF > 2 was considered positive support for *m*_1_ over *m*_0_ (ref. ^58^). We estimated marginal likelihoods using stepping-stone sampling^59^ in BayesTraits^60^. We ran 200 stones with 1 million iterations, drawing values from a beta-distribution (*α* = 0.40, *β* = 1)^59^ and discarding the first 250,000 iterations to ensure convergence. The variable rates model was implemented within an MCMC framework, giving us a posterior distribution of estimated *r* and $\,{\sigma }_{b}^{2}$. Results were replicated over multiple independent chains. All chains were run for a total of 1 billion iterations, sampling every 900,000 iterations after discarding the first 1 million samples. We provide detailed instructions on how to implement these models in the Supplementary Information.

### Characterizing the brain–body mass relationship

We determined the best fitting BBM relationship using Bayes Factors and the equation as described above, comparing (for example) a model with a single overall quadratic (*m*_1_) to a model with multiple slopes (*m*_0_). We identified significant rate heterogeneity in all models (multiple slopes and the curvilinear model). Following previous studies, our multiple-slopes model was constructed to fit a separate slope and intercept for each mammalian order (*N* = 1,436). However, we only did this where we had more than 20 representatives of that order in our data owing to the suggestion that one should have at least 10 data points per parameter estimated^61^ (we estimated a slope and intercept per group, thus we need *N* ≥ 20). The following orders were included in this dataset: Artiodactyla (*N* = 103), Carnivora (*N* = 197), Cetacea (*N* = 45), Chiroptera (*N* = 298), Dasyuromorphia (*N* = 47), Diprotodontia (*N* = 92), Eulipotyphla (*N* = 35), Rodentia (*N* = 349) and Primates (*N* = 227). To maximize sample size, we also included the clade Atlantogenata as a single group (*N* = 43), but for simplicity, we refer to orders when we discuss our results in the context of taxonomic group.

We calculated the proportion of the posterior distribution of each regression parameter that crosses zero (*P*_*x*_). *P*_*x*_ < 0.05 indicates that less than 5% of the posterior distribution crosses zero, and we considered the variable to be substantially different from zero.

To check that our principal results were robust to intraspecific variation, we re-ran the analyses using the dataset of ref. ^5^ (*n*_species_ = 919, *n*_samples_ = 1,908) which sampled multiple (between 1 and 59) brain and body size data per species. Analyses were run using the package MCMCglmm^62^, allowing us to include individual-level variation as well as phylogenetic variance using the median rate-scaled tree from our quadratic model (although results are qualitatively identical when using the time tree). We used alpha-expanded priors on the phylogenetic variance component of the model and default priors on the fixed and residual effects. The results of this model were quantitatively identical to those reported in the main text: a curvilinear model is still strongly supported (*P* < 0.001 for the quadratic parameter, estimated in a Gaussian model with alpha-expanded priors on the phylogenetic variance). We additionally repeated this analysis on a reduced dataset (*n*_species_ = 258, *n*_samples_ = 1,247) that removed singletons, that is, only including species with some level of intraspecific variation, producing qualitatively identical results.

### Directionality in relative brain mass evolution

Our method of detecting rate heterogeneity makes it possible to study evolutionary trends in trait evolution^16^ owing to the fact it introduces biologically meaningful variation into the branch lengths of a phylogeny. Longer branches indicate an increase in the rate of evolution most probably arising from selective forces^36,37^; they have experienced more relative brain mass change than would be expected given their length in time. The sum of all rate-scaled branches along the evolutionary path of a species (‘pathwise rates’) can therefore be used to measure the total amount of adaptive change that species has experienced during its history^16^. We used this logic to determine whether there have been any long-term evolutionary trends in relative brain mass evolution and whether they differ among mammalian orders.

We performed all trends analyses using Bayesian phylogenetic regression. We used the median pathwise rate as our predictor variable (but results do not qualitatively differ when using the mean or mode). We assessed significance of parameters using the *P*_*x*_ < 0.05 criterion described above. All trends analyses were conducted on the median rate-scaled phylogeny to account for differences in the amount of brain mass change expected owing to rate heterogeneity^16^.

### Branchwise magnitudes and proportions of change

To estimate the amount of brain and body mass evolution along each branch of the phylogeny, we used a phylogenetic predictive modelling approach as described in ref. ^16^. This approach allows us to account for not only the relationships we detect here (curvature and trends in brain size) but also rate heterogeneity and a generalized tendency for body mass to increase through time (Cope’s rule)^16^. We first reconstructed body mass at each node of the phylogeny while accounting for the known relationship between body mass and the rate of body mass evolution across mammals^16^. To do this, we ran a ‘variable rates’ model^17^ estimating the rate of body mass evolution across the mammal phylogeny (*N* = 1,504). We then imputed body mass at each node of the phylogenetic tree (see supplementary material for more details on our imputation procedure) using the inferred maximum-likelihood relationship between body mass and the median pathwise rate from this analysis (*β* = 0.009, *α* = 1.07, *P* < 0.001). We then used these body masses to impute ancestral brain mass at each internal node using the median estimated parameters of our BBM plus trends (see ref. ^16^ for details). These reconstructed brain and body sizes provide a realized visualization of our phylogenetic statistical models.

We then tracked rates, body mass change and brain mass change on a branch-by-branch basis across the phylogeny. For each branch, we calculated the magnitude and direction of change for both brain and body mass from start to end. We then estimated the overall proportion of change by dividing ancestral mass by descendant mass, accounting for the time elapsed along the branch.

### Reporting summary

Further information on research design is available in the Nature Portfolio Reporting Summary linked to this article.

### Supplementary information

Supplementary Information Supplementary Information

Reporting Summary

Supplementary Data 1 Data used in our main analysis.

## Figures and Tables

**Extended Data Fig. 2:**
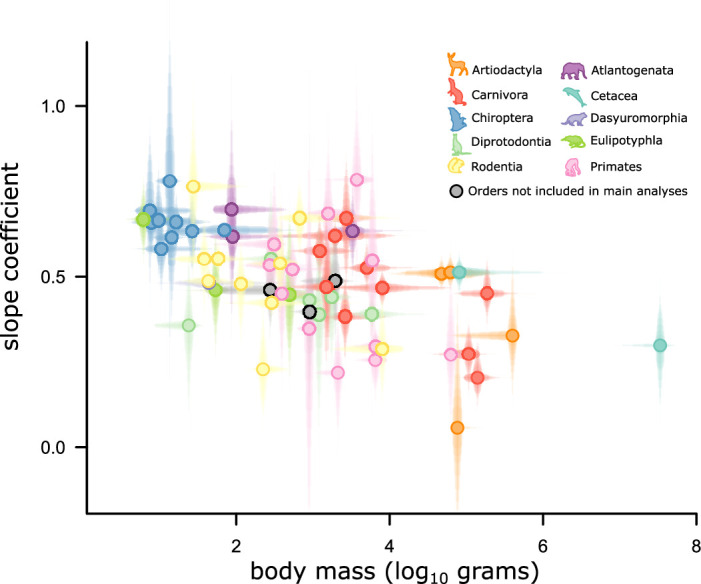
Mammalian family-level slopes. Slope coefficient (and percentiles of posterior distributions plotted as transparent lines) for each mammal family (where N > 5), plotted against body mass (percentiles of body mass range plotted as transparent lines) for each group. Slope coefficients estimated from a multiple-slopes variable rates regression model run in the same way as the multiple-slopes model presented in the main text. Owing to less-stringent size-restrictions (here, we included families where N > 5 to maximize sample size and facilitate comparisons), three families are included which belong to orders not included in our order-level analyses: Didelphidae within Didelphimorphia (N = 17); Peramelidae within Peramelemorphia (N = 11) and Leporidae within Lagomorpha (N = 11).

## Data Availability

All data analysed in the study are provided in the supplementary material.

## Code availability

All analyses in this research were conducted using freely available, published programs and are cited where appropriate in the text.
