# Author Correction: Co-evolutionary dynamics of mammalian brain and body size

**DOI:** 10.1038/s41559-024-02498-2

**Published:** 2024-07-16

**Authors:** Chris Venditti, Joanna Baker, Robert A. Barton

**Affiliations:** 1 School of Biological Sciences, University of Reading, Reading, UK; 2 Department of Anthropology, Durham University, Durham, UK

**Keywords:** Evolution, Phylogenetics, Coevolution

Correction to: *Nature Ecology & Evolution* 10.1038/s41559-024-02451-3, published online 8 July 2024.

In the version of the article initially published, in the first paragraph, the equations now reading “*y* = *ax*^*b*^, where *y* = brain mass, *x* = body mass, *a* = the intercept and *b* = the allometric coefficient” and “log(*y)* = log(*a*) + *b*log(*x*)” originally read “*y* = *bx*^*a*^, where *y* = brain mass, *b* = body mass, *x* = the intercept and *a* = the allometric coefficient” and “log *y* = *a* (log *b* + log *x*)”. Additionally, Extended Data Fig. 3 was missing, while in the last paragraph of the Results, the two callouts to Supplementary Fig. 2 now cite Extended Data Fig. 3. The changes have been made to the HTML and PDF versions of the article.

