# Supplementary material for: Co-evolutionary dynamics of mammalian brain and body size
Source: Nat Ecol Evol. 2024 Jul 8;8(8):1534–42. doi: 10.1038/s41559-024-02451-3 (PMC11310075; doi:10.1038/s41559-024-02451-3)
Supplement: Supplementary file 1 — Supplementary Information [file 41559_2024_2451_MOESM1_ESM.pdf]

nature ecology & evolution

Supplementary information

https://doi.org/10.1038/s41559-024-02451-3

Co-evolutionary dynamics of mammalian brain and body size

In the format provided by the authors and unedited

Contents

Supplementary text

A.1 Identifying evidence for rate heterogeneity in a PGLS model using BayesTraits

A.2 Phylogenetic predictive modelling approach

A.3 Interpretation of the quadratic relationship

Includes table A1

A.4 Assessing the robustness of the quadratic relationship

Includes table A2

Supplementary Figures

A.1 Identifying evidence for rate heterogeneity in a PGLS model using BayesTraits

To determine the amount of rate variability in relative brain size evolution, we use the variable rates regression model (1, 2) implemented within BayesTraits v4 (3). BayesTraits is a stand-alone program that is executed using the command-line.

To run each of the variable rates regression models described in the main text, we input two items into the command-line arguments for BayesTraits. Firstly, we input a tree file in nexus format that includes all species with the relevant data, and secondly we include a tab-delimited text file that contains all the data relevant to testing the specific question at hand in a pre-specified order (species names, y-variable, all x-variables). For example, our single-slope regression model included the following columns: species name (as found in the tree), brain size (our response variable), and body size (our predictor variable). Other analyses are run using the same set of inputs – and full details of how the data file must be structured, along with the nexus file format can be found in the BayesTraits manual available to download from the Reading Evolutionary Biology group website (see specifically pages 8-10): http://www.evolution.rdg.ac.uk/BayesTraitsV3.0.1/Files/BayesTraitsV3.Manual.pdf).

Below is an example of how to execute a regression analysis in BayesTraits using the command-line arguments as described above.

```
BayesTraitsV4.exe treefile.nexus datafile.txt
```

Once the program has been executed, the user is prompted to enter more details about the analysis they wish to perform. For our analyses, we selected option 9) Independent Contrast: Regression (see below). This option is where the variable rates model is implemented and is outlined in full in the BayesTraits manual.

```
Please select the model of evolution to use.
1)      MultiState
4)      Continuous: Random Walk (Model A)
5)      Continuous: Directional (Model B)
6)      Continuous: Regression
7)      Independent Contrast
8)      Independent Contrast: Correlation
9)      Independent Contrast: Regression
12)     Fat Tail
13)     Geo
```

The program then prompts the user to specify whether they would prefer to use maximum likelihood or MCMC. In this case, we were interested in MCMC and so selected option 2.

```
Please select the analysis method to use.
1)    Maximum Likelihood.
2)    MCMC
```

We then manually entered a series of optional model specifications (separated by return) to construct the models as described in our main text. Below, we provide the set of example commands that will run a model with the following structure (as we used in our main analyses):

- Estimates variable rates (see p. 51-52 of the manual).
- Specifies burn-in, sampling period, and total number of iterations (p. 16-17 of the manual).
- Uses stepping-stone sampling (here, we estimate a total of 500 stones with 250,000 iterations per stone, see p.14 for details).
- Draw all parameters relating to rate scalars (both node and branch scalars) from a gamma distribution ($\alpha = 1$). More details on these priors can be found in (2) and (4).

A full list of all possible commands is provided in the manual along with extensive information regarding their use and interpretation.

```
varrates
prior VRBL sgamma 1.10 1.00
prior VRNode sgamma 1.10 1.00
bi 100000000
it 1000000000
sa 900000
stones 500 250000
run
```

Once this model was run, we then used the stand-alone 'Variable Rates Post-Processor' program, available to download from the Reading Evolutionary Biology group website alongside the BayesTraits program itself. This program summarizes the rate scalars estimated along each branch and node; the output of this program was used to create the rate-scaled trees and colour scales in Figure 2, for example.

A.2 Phylogenetic predictive modelling approach

We predicted brain and body size at each node of the mammalian phylogeny Baker et al (5) in order to estimate the amount of brain and body size evolution occurring along individual branches. All predictions were calculated using a maximum-likelihood phylogenetic predictive modelling approach implemented within R (6, 7). We first inferred a maximum-likelihood relationship between body mass and the median-root-to-tip rate from a variable rates model estimating the rate of body mass evolution across the mammal phylogeny limited to include only those species for which we had brain size data (N = 1504). We imputed body mass at each internal node of the phylogeny using the parameters of this relationship ($\beta = 0.009$, $\alpha = 1.07$, $p < 0.001$). We then used the same predictive modelling approach to impute the brain size at each internal node given these body masses and the inferred median parameters from the models we present in the main text. These reconstructions are visualized in figures 4, 5, and Extended Data Figure 1.

A.3 Curvilinearity and the brain-body-size relationship

Whilst we demonstrate that a quadratic parameter is a good approximation of the curvilinearity observed in the mammalian BBM, an alternative representation of the curvilinearity may come in the form of a power model (i.e. even on a log scale the body size may be related to brain size by some power relationship). However, the ability to estimate or quantify exactly what that exponent should be simultaneously alongside a regression relationship, phylogenetic signal, and rate heterogeneity is currently not possible in any standard comparative analytical software. Therefore, we programmatically derived an appropriate power-exponent using a series of systematically varying maximum likelihood models applied to our brain and body size dataset. Specifically, we ran phylogenetic generalised least-squares models of the form $Log\ brain{\sim}Log\ body^{e}$, varying e between 0.1 and 1.5. All models were run in a maximum-likelihood framework implemented within R package caper (6, 8). We repeated the analysis on both the tree where branch lengths were scaled by time and on the median rate-scaled tree from our quadratic variable rates analysis (i.e. the tree depicted in our Figure 2).

We then plotted the likelihood of these models against the modelled exponent (Figure A1), demonstrating a clear peak in likelihood at $e = 0.84$ and $e = 0.77$ for the stretched and time trees, respectively. In both cases, the model with the highest likelihood had a significant relationship with $body^{e}$, strong phylogenetic signal (lambda not significantly different from 1), and the highest R^2 values across the entire model range (0.875 and 0.856 respectively).

We then ran a variable rates regression model estimating a power relationship for both values of e obtained above. As in our main analyses, we ran multiple replicates to ensure convergence. All chains were run for a total of 1 billion iterations, discarding the first 1 million iterations as burn-in and sampling every 900,000 iterations post-convergence. The resulting relationship observed across all mammals is nearly indistinguishable from the quadratic curvature we present in our main results (Figure A1, Figure 1). In addition to the this all subsequent analyses presented in the main text are qualitatively identical if the power curves are used.

Regardless of how exactly we have constructed the curvilinear component of the mammalian BBM, what remains is the exact way to interpret any such observed relationship. In order to break down the estimated parameters of the quadratic relationship we reveal, we calculate – over the full range of observed body size data across mammals – the expected change in brain and body at varying sizes. We do this over the full sample of estimated parameters (Figure A.2 a-d) and create a posterior distribution of the theoretical relationship between brain and body size change and body size itself. To calculate brain and body size change, we use the following equation:

$$\frac{\Delta \log_{10}\mathrm{brain}}{\Delta \log_{10}\mathrm{body}} i = \beta_{1i} + 2\beta_{2i} b,$$

where i is a given iteration of our variable rates regression model, β_1 is the estimated slope parameter (i.e. the linear component of the model) and β_2 is the estimated quadratic parameter (i.e. the curvature), and *b* is a given value of $\log_{10}$ body size.

If there was no curvature, we would expect this relationship to be uniform across the full range of body size – the expected change in brain and body size would be equal for a small-bodied vs. a large-bodied mammal (i.e. equal to the linear component of the model). However, with a negative curvature we can see that large-bodied mammals have proportionally more change in brain mass per unit body mass (both on a $\log_{10}$ scale, Figure A2e). Whilst this applies well over the range of observed body sizes across all mammals, as the curve extends beyond the observe

range, change will ultimately become negative. For a power relationship, whilst we cannot plot the expected change in the same way, we would expect the relationship to asymptote – meaning that beyond some theoretical maximum body size, brain size would no longer increase at all. The exact nature and causality of the curvature of the brain and body size mammals remains an important avenue that needs exploring: revealing the theoretical and empirical underpinning of the curvilinear relationship will be a significant future advance.

The curvilinear model was compared to a multiple-slopes model that estimated a separate slope for each of the major taxonomic groupings within mammals. We assessed model fit using marginal likelihoods and Bayes Factors ($BF,\ BF = -2\log_e[m_1/m_0],$), comparing (for example) the marginal likelihood of our quadratic model (m_1) to the multiple-slopes model (m_0). Where BF > 2, it is considered positive support for m_1 over m_0. The marginal likelihoods from the variable-rate and single-rate models (multiple-slopes and quadratic) are reported in Table A.1.

Table A1. Marginal likelihoods from the models presented in the main text. All models are estimated using PGLS regression implemented within a Bayesian framework (see main methods).

Model	Rate Heterogeneity	Marginal Likelihood
Multiple-slopes	Single-rate	1113.45
Multiple-slopes	Variable-rate	1240.89
Curvilinear (quadratic)	Single-rate	1138.28
Curvilinear (quadratic)	Variable-rate	1318.82

A.4 Assessing curvilinearity across mammalian orders

Our main analysis finds a significant single-slope curvilinear mass-dependent model applied across all mammals. We ensured that this was not being driven by patterns in any one clade by running a series of additional models (one per taxonomic clade) in which we estimated an additional three parameters using contrast coding: an intercept, slope, and quadratic difference. To calculate significance, we obtained the difference in quadratic parameters by assessed the proportion of the posterior distribution of estimated quadratic differences that crossed zero (P_x). Where $P_x < 0.05$, the estimated curvature can be considered distinct from the curvature estimated across all mammals. In all groups, excluding Cetaceans, we found that there was no significant difference between the overall mammalian quadratic and one estimated for the order independently (Table A.2). However, even though Cetaceans were significant, this model was not found to be better supported by the data (BF = 17.35 in favour of the simpler, single quadratic). The same is true for the two other groups for which there is marginal (though non-significant) support for a difference in quadratic in Table S1 – Primates (BF = 12.85) and Atlantogenata (15.94).

The three groups of mammals in which there was any possibility of difference in curvature contain species that have been suggested to be reaching the upper limits of brain sizes (a so-called "gray ceiling") – Proboscideans, Primates, and Cetaceans. To determine that any detected curvature was not being skewed by the largest-bodied (and brained) clade of mammals in terms of absolute size we therefore ran two additional models. In our first model, we excluded all cetaceans. In this model, we find that the quadratic parameter is still highly significant ($p_x = 0$) and of similar magnitude (median $\beta_{quadratic}$ = -0.0193) to that observed across all mammals (median $\beta_{quadratic}$ = -0.0192). This is observed alongside near-identical estimated slope gradients (median β_{slope}= 0.681 without cetaceans vs. median β_{slope}= 0.680 with cetaceans). We then ran an

additional model excluding all Proboscideans, Primates, and Cetaceans with qualitatively identical answers (median β_{slope} = 0.701, median $\beta_{quadratic}$ = -0.024, p_x [for the quadratic parameter] = 0).

Table A2: Parameter differences between a quadratic estimated for each individual taxonomic clades and an overall quadratic. Models estimated using PGLS regression implemented within a Bayesian framework (see main methods). Not all estimated parameters reported here, only the median overall quadratic ($\boldsymbol{\beta_{overall}}$) and the median estimated difference in curvature ($\boldsymbol{\beta_{diff}}$) for each group.

**Group**	$\boldsymbol{\beta_{overall}}$	$\boldsymbol{\beta_{diff}}$	$\mathbf{P_x}$
Artiodactyla	-0.020	0.001	0.483
Atlantogenata	-0.020	0.038	0.051
Carnivora	-0.020	0.003	0.384
Cetacea	-0.020	-0.05	0.016
Chiroptera	-0.011	0.002	0.399
Dasyuromorphia	-0.020	0.011	0.296
Diprotodontia	-0.019	-0.004	0.59
Eulipotyphla	-0.019	-0.035	0.153
Primates	-0.019	-0.037	0.057
Rodentia	-0.023	0.006	0.327

Note that it is important to understand that all these additional analyses are performed in the same way as those in our main analysis. That is, there is underlying rate heterogeneity that is not visible in the raw parameters and likelihoods which can affect apparent significance. We therefore caution over-interpretation of such model comparisons without careful consideration of how rate heterogeneity has been affected.

References

1. Baker J, Meade A, Pagel M, Venditti C. Positive phenotypic selection inferred from phylogenies. Biological Journal of the Linnean Society. 2016;118(1):95-115.
2. Venditti C, Meade A, Pagel M. Multiple routes to mammalian diversity. Nature. 2011;479(7373):393-6.
3. Group REB. BayesTraits V4.1.1 2023 [
4. Pagel M, O'Donovan C, Meade A. General statistical model shows that macroevolutionary patterns and processes are consistent with Darwinian gradualism. Nat Comm. 2022;13(1):1113.
5. Baker J, Meade A, Pagel M, Venditti C. Adaptive evolution toward larger size in mammals. Proceedings of the National Academy of Sciences. 2015;112(16):5093-8.
6. Team RC. R: A Language and Environment for Statistical Computing. R Foundation for Statistical
Computing; 2024.
7. Franks PJ, Freckleton RP, Beaulieu JM, Leitch IJ, Beerling DJ. Megacycles of atmospheric carbon dioxide concentration correlate with fossil plant genome size. Philosophical transactions of the Royal Society of London Series B, Biological sciences. 2012;367(1588):556-64.
8. Orme D, Freckleton RP, Thomas G, Petzoldt T, Fritz SA, Isaac N, et al. caper: Comparative Analyses of Phylogenetics and Evolution in R. R package version 0.5. ed2012.

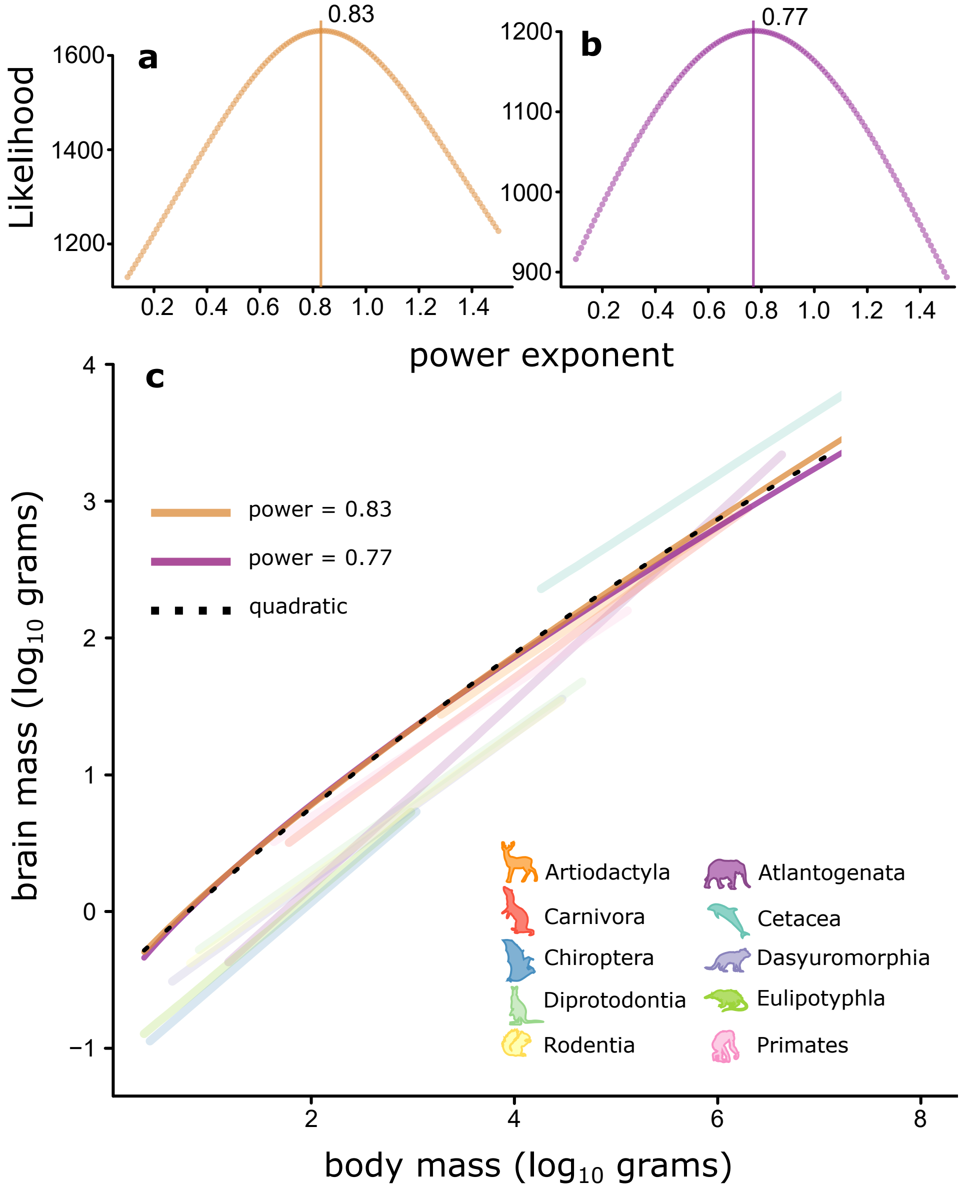

Figure A1: Different types of curvilinearity in the BBM relationship across mammals. *To approximate the appropriate power-scaling to apply to the data, we carried out a series of maximum-likelihood analyses across a range of exponent values. The resulting likelihood distribution is shown for models carried out on the median stretched tree from our main quadratic analysis (a) and on the time tree (b). We then ran a variable rates regression model using these two exponent values as described in the main text; the median plotted power curves for both values (c) are almost indistinguishable from the quadratic relationship we obtain.*

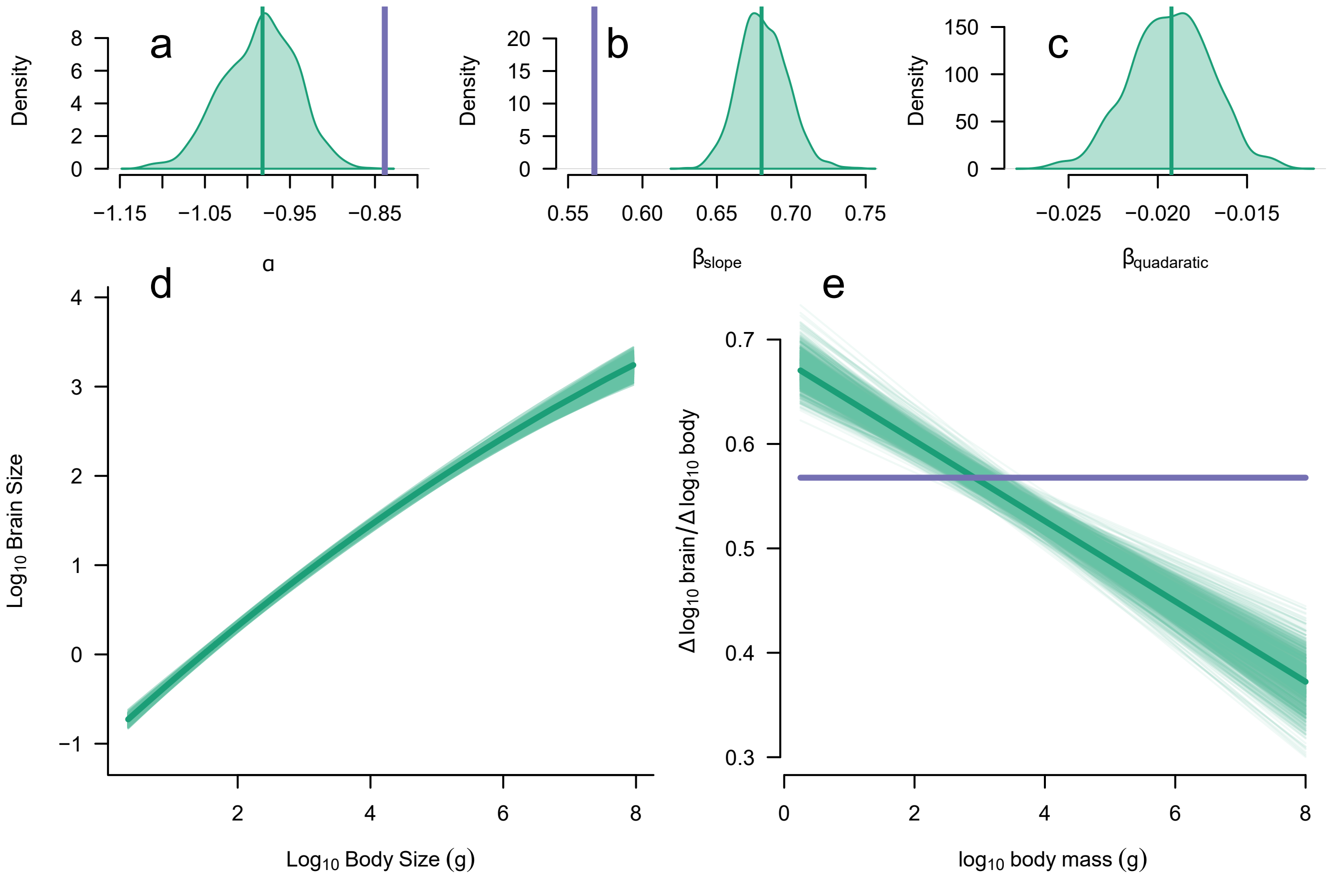

Figure A2: Details and interpretation of the curvilinear brain and body size relationship among mammals. *The posterior distributions of estimated parameters from our curvilinear model are plotted in (a-c), with median values indicated by the vertical solid green line (median α = -0.982, P_x = 0; median β_1 (slope) = 0.68, P_x = 0, and median β_2 (quadratic) = -0.019, P_x = 0. The median parameters from the linear model are indicated by the solid blue lines (median α = -0.838, P_x = 0; median β_1 (slope) = 0.568, P_x = 0). The predicted relationship calculated from the quadratic model parameters are shown in (d), with the median superimposed on top of the posterior distribution of curves. Finally, in (e) we show the expected change in relative brain size ($\frac{\Delta \log_{10} \text{brain}}{\Delta \log_{10} \text{body}}$), calculated over the full range of observed mammalian body sizes. For the linear model (blue line), there is no difference in expected change in relative brain size between a small- and large- bodied mammal.*
